# Bone microarchitecture in patients undergoing parathyroidectomy for management of secondary hyperparathyroidism

**DOI:** 10.1016/j.bonr.2020.100297

**Published:** 2020-07-15

**Authors:** Irene Ruderman, Chamith S. Rajapakse, Angelica Opperman, Patricia L. Robertson, Rosemary Masterson, Mark K. Tiong, Nigel D. Toussaint

**Affiliations:** a Department of Nephrology, The Royal Melbourne Hospital, Parkville, Victoria, Australia; b Department of Medicine (RMH), The University of Melbourne, Parkville, Victoria, Australia; c Departments of Radiology and Orthopaedic Surgery, University of Pennsylvania, PA, USA; d Department of Radiology, The Royal Melbourne Hospital and The University of Melbourne, Parkville, Victoria, Australia

**Keywords:** MRI, magnetic resonance imaging, HRpQCT, high-resolution peripheral quantitative computed tomography, DXA, dual-energy X-ray absorptiometry, BMD, bone mineral density, ALP, alkaline phosphatase, CKD, chronic kidney disease, PTH, parathyroid hormone, SHPT, secondary hyperparathyroidism, Secondary hyperparathyroidism, Renal osteodystrophy, Magnetic resonance imaging, Parathyroidectomy, Chronic kidney disease

## Abstract

**Background:**

Secondary hyperparathyroidism (SHPT) in patients with chronic kidney disease (CKD) leads to complex bone disease, affecting both trabecular and cortical bone, and increased fracture risk. Optimal assessment of bone in patients with CKD is yet to be determined. High-resolution magnetic resonance imaging (MRI) can provide three-dimensional assessment of bone microarchitecture, as well as determination of mechanical strength with finite element analysis (FEA).

**Methods:**

We conducted a single-centre, cross-sectional study to determine bone microarchitecture with MRI in CKD patients with SHPT undergoing parathyroidectomy. Within two weeks of surgery, MRI was performed at the distal tibia and biochemical markers of SHPT (parathyroid hormone [PTH] and alkaline phosphatase [ALP]) were collected. Trabecular and cortical topological parameters as well as bone mechanical competence using FEA were assessed. Correlation of MRI findings of bone was made with biochemical markers.

**Results:**

Twenty patients with CKD (15 male, 5 female) underwent MRI at the time of parathyroidectomy (16 on dialysis, 3 with functioning kidney transplant, one pre-dialysis with CKD stage 5). Median PTH at the time of surgery was 138.5 pmol/L [39.6–186.7 pmol/L]. MRI parameters in patients were consistent with trabecular deterioration, with erosion index (EI) 1.01 ± 0.3, and trabecular bone volume (BV/TV) 10.8 ± 2.9%, as well as poor trabecular network integrity with surface-to-curve ratio (S/C) 5.4 ± 2.3. There was also evidence of reduced cortical thickness, with CTh 2.698 ± 0.630 mm, and FEA demonstrated overall poor bone mechanical strength with mean elastic modulus of 2.07 ± 0.44.

**Conclusion:**

Patients with severe SHPT requiring parathyroidectomy have evidence of significant changes in bone microarchitecture with trabecular deterioration, low trabecular and cortical bone volume, and reduced mechanical competence of bone.

## 1 Introduction

Bone abnormalities seen in patients with chronic kidney disease (CKD) complicated by secondary hyperparathyroidism (SHPT) result in impaired bone quality and quantity, affecting both trabecular and cortical bone compartments. SHPT leads to abnormal trabecular connectivity, cortical thinning, and decreased bone mineral density (BMD) (Amling et al., 1994; Parfitt, 1998) which increase fracture risk. Fractures are significantly more prevalent in patients with CKD and SHPT and are associated with increased hospitalisations, mortality and morbidity (Alem et al., 2000).

Assessment of bone quality is challenging in patients with CKD as standard serological markers of bone turnover do not reliably distinguish between different renal bone pathologies and, in addition, commonly used biomarkers can have assay limitations and inconsistent results in the CKD population (Coco and Rush, 2000; Sprague et al., 2016; Danese et al., 2006). Dual-energy X-ray absorptiometry (DXA) is widely used in the general population to diagnose and monitor low BMD as well as provide fracture risk assessment (Kanis, 2002). In the general population there is a strong association between low areal BMD and increased risk of fracture (Marshall et al., 1996). However, DXA has significant limitations in the CKD population and can potentially overestimate vertebral BMD due to the presence of overlying aortic calcification. In recent years, several prospective cohort studies have demonstrated utility of DXA in assessing fracture risk in patients with CKD stages 3–5 (Yenchek et al., 2012; West et al., 2015; Iimori et al., 2011) and DXA is now recommended in the updated 2017 Kidney Disease Improving Global Outcomes (KDIGO) clinical guidelines to determine BMD and evaluate osteoporosis or renal bone disease in patients with CKD and those on dialysis (Ketteler et al., 2017). DXA, however, provides poor distinction between cortical and trabecular bone, as it is a two-dimensional (2D) assessment of BMD.

Bone biopsy remains the gold standard for adequately diagnosing renal bone disease but is rarely performed as biopsy acquisition is invasive, challenging to process and analyse, and fails to permit longitudinal measurements of bone structure at the same location. Bone biopsy is often performed at the iliac crest, a region predominantly composed of trabecular bone with low load and low fracture prevalence. SHPT is associated with loss of predominantly cortical bone in long bones (Osima et al., 2018), which are the commonest site of fracture, therefore bone biopsy of the iliac crest may not provide adequate prognostic information on clinical outcomes. Optimal assessment of renal bone disease is yet to be determined, however new imaging methods may be able to quantitatively assess and monitor bone fragility *in vivo*, including high-resolution magnetic resonance imaging (MRI, or micro-MRI) and high-resolution peripheral quantitative computed tomography (HR-pQCT).

Micro-MRI is a non-invasive technique that provides three-dimensional (3D) assessment of bone and can be used for repeat monitoring without exposure to ionising radiation as with HR-pQCT. Micro-MRI of bone trabecular architecture was first described 20 years ago (Jara et al., 1993) and since then technical advances in image acquisition and analysis have seen its performance rival that of HR-pQCT. Micro-MRI provides high-resolution imaging of bone allowing the evaluation of both cortical and trabecular properties at a scale of 100–200 μm (in-plane resolution) (Singh et al., 2018). Assessment of bone volume fraction and bone topology correlates well with equivalent computed tomography (CT) measurements (Krug et al., 2008).

In addition to analysis of bone microarchitecture, micro-MRI images can also be used for finite element analysis (FEA), which provides a more direct assessment of the mechanical competence of bone. FEA is based on mechanical engineering technology which facilitates virtual stress testing of bone to compute metrics of bone mechanical competence, specifically providing information on bone stiffness and elastic modulus (Chang et al., 2014). In the CKD population, micro-MRI has been used to study bone microarchitecture in patients on dialysis and those pre- and post-kidney transplantation (Wehrli et al., 2004; Sharma et al., 2018; Rajapakse et al., 2012; Link et al., 2002; Leonard et al., 2019; Rajapakse et al., 2017). In a group of 17 patients on haemodialysis (HD), micro-MRI was used to quantify trabecular and cortical structural parameters, which are traditionally evaluated with bone biopsy (Wehrli et al., 2004), compared to an age- and sex-matched control group and results were consistent with increased bone fragility in HD patients compared to the control group.

To date, micro-MRI to evaluate bone microarchitecture associated with severe SHPT in patients prior to parathyroidectomy has not been reported. A previous study using DXA in a cohort of patients following parathyroidectomy, demonstrated increased BMD post-surgery (Fang et al., 2018), however this was based on retrospective data over a four-year period. HR-pQCT has been evaluated in patients with primary hyperparathyroidism undergoing a parathyroidectomy with studies reporting significant improvement in total, cortical, and trabecular volumetric bone density as early as 6 months post parathyroidectomy, as well as improved FEA stiffness and failure load (Hansen et al., 2012). Micro-MRI in patients with SHPT undergoing surgery may provide more detailed information regarding bone microarchitecture and bone strength compared to other current imaging techniques. This imaging technology has the capacity to distinguish patients who have evidence of bone disease related to high PTH and those who may not have significant structural bone disease despite high PTH. This information could be valuable to determine who may benefit from parathyroidectomy or who may not, or even who might potentially suffer adverse consequences from parathyroidectomy with risk of developing adynamic bone disease. Static MRI parameters, unlike bone biopsy, provide a representation of the cumulative impact on bone structure due to changes in bone turnover over time.

The landscape of management for SHPT in Australia significantly changed in August 2015 following withdrawal of government reimbursement for the calcimimetic agent cinacalcet. Over the past five years there has been an increase in the number of patients with severe and progressive SHPT requiring surgical management with a parathyroidectomy, as medical treatment with cinacalcet was not as readily available and other therapies for SHPT, such as calcitriol, are not always effective at suppressing PTH levels. At our institution, the number of parathyroidectomies performed doubled from 2015 to 2019 following withdrawal of funding for cinacalcet. Establishing a non-invasive method to evaluate and monitor underlying bone pathology in SHPT is essential and will assist in future management of this challenging problem, particularly in comparing the effectiveness of medical *versus* surgical SHPT treatment. The aim of this study was to determine micro-MRI parameters of bone microarchitecture in patients with SHPT undergoing parathyroidectomy and correlate MRI findings of bone disease with biochemical markers of SHPT at the time of surgery.

## 2 Methods

### 2.1 Study participants

Patients with CKD undergoing planned surgical parathyroidectomy for SHPT at The Royal Melbourne Hospital between January 2019 and February 2020 were approached to participate in this single-centre, cross-sectional study. Patients over the age of 18 and able to provide informed consent with no contraindication to MRI were included in the study. A detailed medical history and MRI safety questionnaire was recorded at pre-admission clinic prior to MRI scanning. The study was approved by the Melbourne Health Human Research Ethics Committee (#HREC2019.029) and was conducted in accordance with the Declaration of Helsinki.

### 2.2 MRI

Micro-MRI of the distal tibia was performed within two weeks of parathyroidectomy with a commercial 3.0-Tesla whole-body imager (Siemens Trio, Erlangen, Germany). Participants were imaged in a feet-first prone position. MRI acquisitions were performed at the distal tibial metaphysis using a 3D-turbo spin echo pulse sequence (Flip angle 180, repetition time/echo time 53/16 ms, field-of-view 70 mm × 70 mm, voxel size 0.273 × 0.273 × 0.6 mm^3^, 12 signal averages, and echo train length of two). This sequence is commercially available on clinical MRI systems with a scan time of 12 min. The MRI scan was undertaken utilising a commercially available 15 channel transmit receive knee coil (Siemens, Erlangen, Germany). The centre of the coil was positioned so that images were obtained 1 cm proximal to the midpoint of the medial malleolus.

Raw MRI data was pre-processed *via* bone volume fraction (BVF) mapping and segmentation of the bone into trabecular and cortical compartments for structural measurements and topological analysis using published algorithms (Rajapakse et al., 2012; Wehrli, 2007). Data analysis was performed by two study investigators (CR, AO) offsite at the University of Pennsylvania, PA, USA. Segmentation was determined by delineating the periosteal and endosteal boundaries of the bone with an operator-guided semi-automatic algorithm using custom-built software (Rajapakse et al., 2012) and standard microarchitecture parameters were derived for trabecular and cortical bone. Table 1 outlines MRI parameters evaluated in this study.

**Table 1:** Description of MRI parameters evaluated in this study.

MRI parameter	Description
Surface to curve ratio (S/C)	Indicates the plate (surface) to rod (curve) ratio of the trabeculae. A higher ratio is a marker of greater trabecular network integrity (Rajapakse et al., 2014).
Erosion index (EI)	Represents topological parameters expected to increase *versus* those expected to decrease, with erosion cause by osteoclastic resorption. A higher index is consistent with greater trabecular deterioration (Rajapakse et al., 2014).
Bone volume/total volume (BV/TV) (%)	Represents the ratio of bone volume to total volume in the region of interest.
Trabecular thickness (TbTh) (mm)	Represents mean thickness of trabeculae
Trabecular number (TbN) (1/mm)	Represents mean number of trabecular per unit length
Trabecular separation (TbS) (mm)	Represents the bone marrow space between trabeculae
Cortical thickness (CTh) (mm)	Represents mean cortical bone thickness
Elastic modulus (GPa)	Main component of FEA and a marker of bone mechanical competence

### 2.3 FEA

FEA allows for *in vivo* estimation of bone mechanical properties. Three-dimensional micro-finite element models generated from acquired images of the distal tibia were subjected to stimulated loading in inferior-superior direction, to compute the elastic modulus of the entire cross-section of the bone (Chang et al., 2017; Rajapakse et al., 2009; Rajapakse et al., 2014; Rajapakse et al., 2010). This data can be interpreted as bone mechanical competence or strength – the higher the elastic modulus, the greater the strength of the bone.

### 2.4 Biochemical measurements

Serum was collected in all patients prior to parathyroidectomy (within two weeks of surgery). Samples were collected to measure the following parameters: PTH, calcium, phosphate, alkaline phosphatase (ALP), C-reactive protein (CRP), and albumin. Serum calcium level was adjusted as follows if serum albumin was <40 g/L: corrected serum calcium (mmol/L) = measured serum calcium (mmol/L) + 0.02 (40 - serum albumin (g/L)).

### 2.5 Statistical analysis

Results are presented as mean (±standard deviation) for normally distributed variables and as median (and interquartile range) for variables with non-parametric distribution. Relationships were studied using Pearson or Spearman correlation, depending on the distribution of variables. Two-tailed p values < 0.05 were considered statistically significant. All statistical analyses were performed using SPSS version 21.0 for Macintosh (SPSS, Chicago, IL). Graphics were created with GraphPad Prism 8 for Macintosh (La Jolla, CA, USA).

## 3 Results

### 3.1 Demographics and biochemical outcomes

Twenty-three patients underwent surgical parathyroidectomy at The Royal Melbourne Hospital from January 2019 to February 2020. All patients were approached to participate in the study. Three patients were excluded due to MRI being contraindicated (claustrophobia, n = 2; foreign material on orbital X-ray, n = 1). Twenty patients were enrolled in the study, with micro-MRI performed within two weeks of surgery (2.5 [1.3–4] days). Sixteen patients were on dialysis, three patients had a functioning kidney transplant (mean time post-transplant 14 ± 5 months) and one patient was pre-dialysis with CKD stage 5. Mean serum creatinine for patients with a kidney transplant was 90 ± 35 μmol/L and the patient with CKD stage 5 had a serum creatinine of 560 μmol/L. All patients had biochemical evidence of SHPT and were therefore included in the study to maximise study sample size. Participant demographics are outlined in Table 2 and biochemistry outlined in Table 3.

**Table 2:** Demographics and clinical characteristics.

Demographics	Participants (n = 20)
Age, years	48 ± 12
Gender, male	15
Stage of CKD and/or dialysis modality	
- CKD stage 5 (non-dialysis)	1
- HD (satellite)	9
- HD (home)	2
- PD	5
- Kidney transplant recipient	3
Time on dialysis, years (n = 16)	2.9 [1–5]
Aetiology of CKD	
- Diabetic nephropathy	2
- Glomerulonephritis	9
- Reflux nephropathy	4
- Other	5
Co-morbidities	
- Previous parathyroidectomy	1
- Previous fracture	3
- Cinacalcet use in past 6 months	0
- Diabetes mellitus	4
- Cardiovascular disease	5
- Hypertension	13
- History of osteoporosis	1
- Failed transplant	6
- Smoker/ex-smoker	6

Data presented as number, mean ± standard deviation or median [interquartile range].

Abbreviations: CKD, chronic kidney disease; HD, haemodialysis; PD, peritoneal dialysis;

**Table 3:** Biochemistry at time of parathyroidectomy.

Biochemistry	Participants (n = 20)	Normal range
PTH, pmol/L	138.5 [39.6–186.7]	1.7–10.0
Adjusted calcium, mmol/L	2.5 ± 0.2	2.1–2.6
Phosphate, mmol/L	1.7 ± 0.6	0.75–1.50
ALP, IU/L	176 [103–274]	30–120
CRP mg/L	2 [1–5]	<3
Albumin g/L	31 ± 1.5	32–45

Data presented as mean ± standard deviation or median [interquartile range].

Abbreviations: ALP, alkaline phosphatase; CRP, C-reactive protein; PTH, parathyroid hormone.

### 3.2 MRI findings

**Fig. 1:**
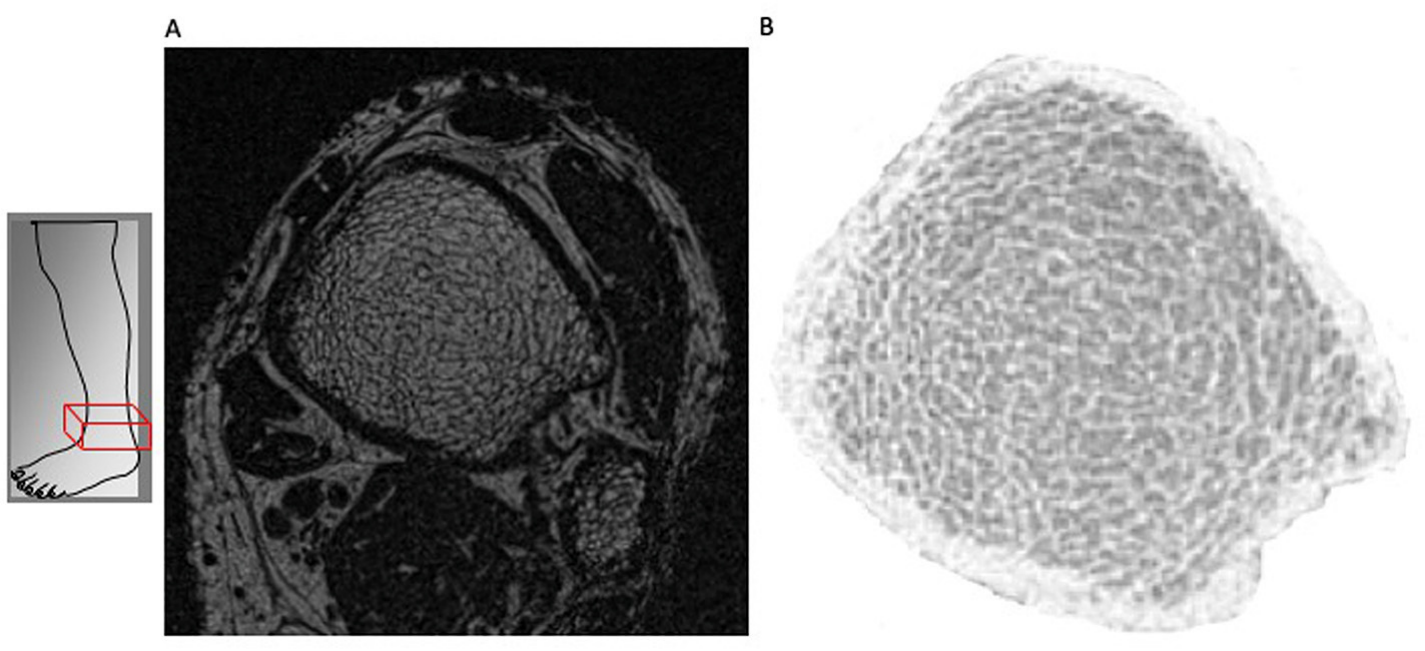
A: Anatomic site of MRI image of distal tibia and fibula. B: High-resolution MRI image through distal tibia showing trabecular and cortical microarchitecture.

MRI of participants showed trabecular bone volume (BV/TV) of 10.8 ± 2.9% and surface-to-curve ratio (S/C) of 5.4 ± 2.3. Representative MRI images illustrating trabecular and cortical compartments of a study participant are shown in Fig. 1. Mean values of trabecular and cortical structural parameters assessed at the distal tibia by MRI in the whole cohort, as well as divided into combined dialysis patients and pre-dialysis CKD stage 5 patient (n = 17) compared with patients with a functioning kidney transplant (n = 3), and by gender (15 male, 5 female), are presented in Table 4, Table 5 respectively. There were no statistically significant differences in trabecular or cortical parameters between CKD stage 5 patients (dialysis and non-dialysis) compared to patients with a kidney transplant. Female patients with SHPT had lower trabecular bone volume (BV/TV, 9 ± 21.1% *versus* 11.5 ± 3%, p = 0.04) and trabecular thickness (TbTh, 0.12 ± 0.002 mm *versus* 0.13 ± 0.008 mm, p = 0.01) compared to male patients, there was no difference in cortical thickness between genders (p = 0.17). Table 6 outlines MRI parameters at the distal tibia in previously published cohorts compared to our current study cohort. Micro-MRI remains an emerging imaging modality therefore no normal reference ranges for the distal tibia exist at present. As a result, micro-MRI parameters from two publications with non-CKD patients (Wehrli et al., 2004; Benito et al., 2003) are used as predicted normal ranges.

**Table 4:** MRI parameters in patients with SHPT at time of parathyroidectomy.

MRI parameter	All patients (n = 20)	CKD stage 5 patients (n = 17; dialysis n = 16, non-dialysis, n = 1)	Renal transplant patients (n = 3)	p value
S/C	5.4 ± 2.3	5.3 ± 2.4	5.2 ± 1.5	0.96
EI	1.01 ± 0.3	1.02 ± 0.3	0.98 ± 0.27	0.93
BV/TV (%)	10.8 ± 2.9	10.9 ± 3	10.4 ± 2.7	0.88
TbTh (mm)	0.13 ± 0.007	0.128 ± 0.007	0.127 ± 0.006	0.86
TbN (1/mm)	0.84 ± 0.17	0.84 ± 0.16	0.81 ± 0.17	0.89
TbS (mm)	1.11 ± 0.22	1.09 ± 0.2	1.16 ± 0.3	0.89
CTh (mm)	2.7 ± 0.63	2.8 + 0.63	2.3 ± 0.5	0.46

Data presented as mean ± standard deviation.

Abbreviations: S/C, surface to curve ratio; EI, erosion index; BV/TV (%), trabecular bone volume; TbTh (mm), trabecular thickness; TbN (1/mm), trabecular number; TbS (mm), trabecular separation; CTh (mm), cortical thickness.

**Table 5:** MRI parameters in patients with SHPT at time of parathyroidectomy based on gender.

MRI parameter	Male (n = 15)	Female (n = 5)	p value
S/C	5.8 ± 2.4	3.9 ± 0.77	0.06
EI	0.9 ± 0.3	1.2 ± 0.26	0.14
BV/TV (%)	11.5 ± 3	9 ± 21.1	**0.04**
TbTh (mm)	0.13 ± 0.008	0.12 ± 0.002	**0.01**
TbN (1/mm)	0.88 ± 0.17	0.72 ± 0.08	0.08
TbS (mm)	1.05 ± 0.2	1.3 ± 0.2	0.08
CTh (mm)	2.8 ± 0.66	2.3 ± 0.3	0.17
Elastic modulus (GPa)	2.04 ± 0.43	2.12 ± 0.47	0.8

Data presented as mean ± standard deviation.

Abbreviations: S/C, surface to curve ratio; EI, erosion index; BV/TV (%), trabecular bone volume; TbTh (mm), trabecular thickness; TbN (1/mm), trabecular number; TbS (mm), trabecular separation; CTh (mm), cortical thickness.

Bold data indicate significance at p value < 0.05.

**Table 6:** Comparison of demographic and distal tibial MRI parameters to published studies.

Parameter	Study cohort (n = 20)	Benito et al. (2003) (n = 10)	Benito et al. (2003) (n = 10)	Sharma et al. (2018) (n = 14)	Wehrli et al. (2004) (n = 17)	Wehrli et al. (2004) (n = 17)
Population	Dialysis patients with SHPT	Eugonadal men	Hypogonadal men	CKD pre kidney transplant	HD patients	Control group
Age	48 ± 12	53.7 ± 13.2	53.1 ± 13.4	46 ± 11.3	40.3 ± 6.4	40.2 ± 6.7
Gender, male	15 (75%)	10 (100%)	10 (100%)	9 (62.5%)	9 (53%)	9 (53%)
CKD stage	CKD5 on dialysis	No CKD	No CKD	CKD pre transplant	CKD5 on dialysis	No CKD
S/C	5.4 ± 2.3	10.8 ± 2.4	6.9 ± 1.8	5.51 ± 1.36	5.6 ± 1.5	6.4 ± 0.6
EI	1.01 ± 0.3	0.89 ± 0.13	1.21 ± 0.24	0.91 ± 0.25	1.17 ± 0.45	0.97 ± 0.21
BV/TV (%)	10.8 ± 2.9	14.3 ± 1.1	12 ± 1.6	10.4 ± 2.2	12 ± 2.2	13.3 ± 2
TbTh (mm)	0.128 ± 0.007			0.126 ± 0.005	0.151 ± 0.019	0.156 ± 0.013
TbN (1/mm)	0.84 ± 0.17			0.82 ± 0.14		
TbS (mm)	1.107 ± 0.219			1.120 ± 0.023		
CTh (mm)	2.698 ± 0.630			2.632 ± 0.545		

Abbreviations: S/C, surface to curve ratio; EI, erosion index; BV/TV (%), trabecular bone volume; TbTh (mm), trabecular thickness; TbN (1/mm), trabecular number; TbS (mm), trabecular separation; CTh (mm), cortical thickness; CKD, chronic kidney disease.

### 3.3 Correlation between biochemical variables and structural MRI parameters

**Fig. 2:**
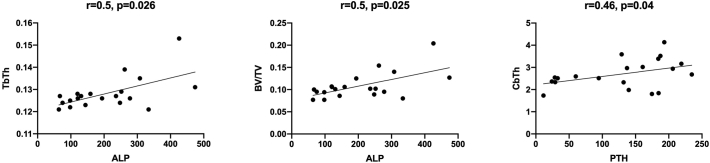
Spearman correlations (r) between trabecular and cortical topological parameters derived by MRI and bone turnover markers (PTH and ALP).

There was a weak correlation between ALP and trabecular parameters specifically BV/TV (r = 0.5, p = 0.025) and TbTh (r = 0.5, p = 0.026) (Fig. 2). Unlike previous published literature by Sharma et al. (2018), which showed that PTH correlated with both S/C and erosion index (EI), in our study there was only a correlation between PTH and cortical thickness (r = 0.46, p = 0.04) (Fig. 2). Spearman correlations are outlined in Table 7.

**Table 7:** Spearman correlations (r) between trabecular and cortical microarchitecture parameters (determined by MRI) and bone turnover markers.

MRI parameter	ALP	PTH
S/C	0.34	0.23
EI	−0.32	−0.12
BV/TV (%)	0.5⁎	0.27
TbTh (mm)	0.5⁎	0.32
TbN (1/mm)	0.44	0.22
TbS (mm)	−0.44	−0.22
CTh (mm)	0.1	0.46⁎

Abbreviations: S/C, surface to curve ratio; EI, erosion index; BV/TV (%), trabecular bone volume; TbTh (mm), trabecular thickness; TbN (1/mm), trabecular number; TbS (mm), trabecular separation; CTh (mm), cortical thickness.

⁎ p ≤ 0.05.

### 3.4 FEA results

Elastic modulus in the study cohort ranged between 1.31 and 2.80 (mean 2.07 ± 0.44). Two patients with a history of calcaneal and neck of femur fractures had low elastic modulus of 1.31 and 1.65 respectively. There was a negative correlation between age and elastic modulus (r = −0.48, p = 0.03), and there was no correlation between dialysis vintage or gender and elastic modulus.

## 4 Discussion

This study examines bone microarchitecture of the distal tibia in patients with CKD and SHPT at the time of parathyroidectomy using the novel imaging technique of micro-MRI. Key findings include significant trabecular topological abnormalities with low S/C ratio, BV/TV and elevated EI consistent with greater trabecular deterioration and lower trabecular integrity and bone volume in patients with SHPT compared to published cohorts of individuals with no kidney disease. There was also evidence of reduced cortical thickness and low elastic modulus in keeping with poor bone mechanical competence. Although there were statistically significant correlations with ALP, PTH and some trabecular and cortical microarchitecture parameters, these associations were weak and highlight the imprecision of using bone turnover markers alone to identify and monitor renal bone disease.

Renal osteodystrophy is commonly seen in patients with CKD stage 5 on HD (Trombetti et al., 2013), with bone abnormalities contributing to increased fracture risk (Yenchek et al., 2012) as well as an associated increased risk of vascular calcification (Moldovan et al., 2011). The majority of studies evaluating BMD and fracture in patients with CKD have been performed using DXA or HR-pQCT. West et al. demonstrated in pre-dialysis CKD patients that hip BMD, calculated by either DXA or HR-pQCT, was significantly lower in those with a history of incident fractures compared to those without (Yenchek et al., 2012). A major concern with DXA in the CKD population is the inability to accurately analyse bone microstructure. Differentiation between cortical and trabecular bone is very challenging and can be influenced by artefactual vascular calcification projections particularly at the lumbar spine.

Alterations of bone microarchitecture contribute to skeletal fragility, independent of areal BMD, and are equally important to evaluate fracture risk (Boutroy et al., 2005; Sornay-Rendu et al., 2007). Negri et al. imaged the distal radius and tibia with HR-pQCT in patients on HD and showed that significantly deceased cortical and trabecular parameters correlated with severity of SHPT in women (Negri et al., 2012). Of note, the medium serum intact PTH was 63 pmol/L [4.3–295.5] in this group, which is much lower than the median serum PTH in our current study participants (138.5 pmol/L [39.6–186.7]). Elevated PTH has previously been associated with reduced cortical volumetric BMD, increased cortical porosity and reduced cortical thickness (Osima et al., 2018). Our study found a weak positive correlation between PTH and cortical thickness (r = 0.46, p = 0.04). This was a surprising finding as we would have expected to see a negative association between PTH and cortical thickness, and larger studies using micro-MRI are required to explore this association further. Although PTH is routinely used to monitor SHPT in patients with CKD, it is not a maker of bone turnover *per se*, as it does not accurately reflect osteoblastic or osteoclastic action in the bone. Additionally, not all circulating PTH in patients with advanced CKD is biologically active, with a proportion of PTH undergoing oxidation thereby losing biological activity (Hocher et al., 2013), and circulating PTH has an extremely short half-life (minutes), therefore PTH levels fluctuate significantly and a trend rather than a single value is more useful to monitor SHPT progression. PTH alone is also insufficient to differentiate the type of renal bone disease present, as suggested by the weak correlations in the study cohort.

Similar findings to our study were identified in a study of 74 patients on HD (mean PTH 355 pg/mL), with particularly female dialysis patients having markedly impaired bone microarchitecture when assessed by HR-pQCT compared to a gender- and race-matched healthy control group (Cejka et al., 2011). Addition of FEA to bone microarchitecture analysis of the distal radius and tibia by HR-pQCT was evaluated by Trombetti et al. (2013) in patients on HD and findings were consistent with previous studies. Addition of FEA, unsurprisingly, showed that stiffness and predicted load failure were lower at both the distal radius and tibia in women with CKD but not in men, compared with age-matched controls.

Few studies have assessed the utility of micro-MRI in evaluating bone microarchitecture in CKD patients. Sharma et al. (2018) compared bone microarchitecture assessment using DXA, pQCT and micro-MRI with ‘gold standard’ bone biopsy in 14 patients with CKD prior to undergoing surgery (13 kidney transplantation, one parathyroidectomy). Micro-MRI changes in this study correlated well with bone histomorphometry and DXA, with significant correlations observed between histomorphometric mineralisation and turnover indices and various MRI parameters. MRI-derived trabecular parameters were also significantly related to femoral neck BMD. Importantly, micro-MRI parameters were comparable to results in our study and corroborate findings of severe renal osteodystrophy in the current cohort, both in dialysis patients and a small sample of kidney transplant recipients, with bone loss and deranged microarchitecture in relatively young and mostly male patients. Another study of high-resolution MRI in patients with CKD was performed by Link et al. (2002), investigating structural measures of the calcaneus compared to BMD in the lumbar spine using DXA in 60 kidney transplant recipients, pre- and post-transplantation. This study reported significantly lower BV/TV, TbN, TbTh and a higher TbS, together with significantly lower BMD of the lumbar spine in patients with prevalent fractures compared to those without.

Micro-MRI has emerged as a novel tool to accurately and safely assess both trabecular and cortical bone without risk of radiation exposure. The ability to incorporate FEA provides useful information regarding bone strength which could be used to monitor treatment effect. Micro-MRI was used in a cohort of hypogonadal and eugonadal males to assess trabecular architecture of the distal tibia (Benito et al., 2003). Benito et al. showed that the S/C ratio was 36% lower (p = 0.004) and the EI was 36% higher (p = 0.003) in hypogonadal men, suggesting that male hypogonadism was associated with marked deterioration of trabecular architecture. When comparing these results to male patients in the current study, our patients with severe SHPT have lower S/C ratio and BV/TV than both hypogonadal and eugonadal males with normal kidney function. Testosterone levels were not measured in the study population, although it is likely that a significant proportion of male patients would have hypogonadism given the prevalence of this endocrinological issue in kidney disease is reported to be greater than 50% (Albaaj et al., 2006; Gungor et al., 2010).

Peak BMD or BV/TV is a genetic predisposition influenced by multiple environmental factors and women typically have a lower peak BV/TV compared to males. In an exclusively female population, micro-MRI was used to assess trabecular structural bone parameters of the distal radius in a postmenopausal osteopaenic cohort pre- and post-treatment with bisphosphonates (Folkesson et al., 2011; Greenspan et al., 2010). To date, however, there is no published literature of bone microarchitecture parameters in premenopausal women with normal kidney function. In our study there were statistically significant reductions in both BV/TV and TbTh in females compared to males with SHPT, despite no significant differences in age or dialysis vintage.

Similar to the study by Benito et al., FEA analysis of the distal tibia with micro-MRI was performed in another cohort of eugonadal and hypogonadal males to assess the effects of testosterone treatment. Zhang et al. (2008) showed improved elastic moduli of the tibial trabecular bone by increased trabecular plate thickness following testosterone therapy in hypogonadal males. These studies highlight the usefulness of FEA derived from micro-MRI for monitoring treatment response. FEA using specialised MRI algorithms have been validated for the distal tibia (Rajapakse et al., 2018) and more recently for the femur (Rajapakse and Chang, 2018; Rajapakse et al., 2020). FEA of the femur is especially useful for evaluating fracture risk in the hip as it can simulate forces experienced during a sideways fall, which accounts for the main orientation in which hip fractures occur (Keyak et al., 2011). Using FEA derived from micro-MRI images in patients with SHPT could enable targeted therapies to be comprehensively evaluated with the effect on bone microarchitecture, and ultimately risk of fracture, established.

There are some limitations to our study including the small sample size and cross-sectional nature of the study which does not allow assessment of bone changes over time and evaluation as to whether surgical treatment may lead to improvement in bone microarchitecture. A 12-month follow up study is planned for our cohort of study patients to assess this question however, where each patient can be used as their own control. Another limitation is the lack of a matched dialysis population without severe SHPT. There was also no concurrent DXA scan evaluation performed, although the predictive validity of fracture of this imaging modality in dialysis patients has previously been described in the literature (Iimori et al., 2011). One strength of our study is the use of a commercially available, routinely used RF coil and sequence acquisition for MRI scanning, which will hopefully allow this imaging modality to be more widely accessible. Also, the use of FEA in this patient cohort is novel and likely to become a tool for monitoring treatment in the future.

## 5 Conclusion

In conclusion, using a novel, non-invasive and ionising-radiation free imaging modality, patients with severe SHPT prior to parathyroidectomy showed evidence of significant bone microarchitecture changes with trabecular deterioration, reduced trabecular bone integrity, low trabecular and cortical bone volume, and reduced mechanical competence of bone as identified on FEA. Commonly used bone turnover markers in CKD, including PTH and ALP, correlated poorly with trabecular and cortical topological parameters and confirm the need for high quality imaging to be used as a diagnostic tool in renal bone disease. The use of micro-MRI in this population has the potential to guide treatment strategies in the future.

## Declaration of competing interest

The authors declare that they have no known competing financial interests or personal relationships that could have appeared to influence the work reported in this paper.

NDT has received honoraria, travel support and research funding from Amgen, Shire and Sanofi. PLR has received honoraria and travel support Takeda, Sanofi and Pfizer.
